# Clinical and Pathological Characteristics of Hyaline-Vascular Type Unicentric Castleman Disease: A 20-Year Retrospective Analysis

**DOI:** 10.3390/diagnostics11112008

**Published:** 2021-10-28

**Authors:** Midori Filiz Nishimura, Yoshito Nishimura, Asami Nishikori, Yukina Maekawa, Kanna Maehama, Tadashi Yoshino, Yasuharu Sato

**Affiliations:** 1 Department of Pathology, Okayama University Graduate School of Medicine, Dentistry, and Pharmaceutical Sciences, Okayama 700-8558, Japan; p2hq21br@s.okayama-u.ac.jp (M.F.N.); yoshino@md.okayama-u.ac.jp (T.Y.); 2 Department of General Medicine, Okayama University Graduate School of Medicine, Dentistry, and Pharmaceutical Sciences, Okayama 700-8558, Japan; nishimura-yoshito@okayama-u.ac.jp; 3 Department of Medicine, John A. Burns School of Medicine, University of Hawai’i, Honolulu, HI 96813, USA; 4 Division of Pathophysiology, Okayama University Graduate School of Health Sciences, Okayama 700-8558, Japan; asami.kei@s.okayama-u.ac.jp (A.N.); paky6h76@s.okayama-u.ac.jp (Y.M.); pz4g0ufl@s.okayama-u.ac.jp (K.M.)

**Keywords:** Castleman disease, hyaline vascular type, unicentric Castleman disease, abdominal cavity, mediastinum

## Abstract

The first case of hyaline vascular type of unicentric Castleman disease (HV-UCD) was reported more than six decades ago. Since patients with HV-UCD are often asymptomatic and this condition is generally discovered incidentally on imaging tests, most of the previous reports were of mediastinal origin detected by chest radiography. In recent years, improved access to imaging modalities has provided new insights in the diagnosis of this condition. In this study, we reviewed the detailed clinical and pathological findings of 38 HV-UCD cases (20 males and 18 females, mean age: 42.8 years). The most common site involved was the abdominal cavity (34.2%), followed by mediastinum (23.7%) and retroperitoneum (15.8%). In the abdominal cavity, mesenteric origin was the most common. The mean size of masses was 4.8 cm. Pathologically, thick hyalinized collagen fibers surrounding large blood vessels and calcification were observed (81.6% and 23.7%, respectively). Multinucleated giant cells resembling Warthin–Finkeldey cell were also observed in occasional cases (23.7%). This is a unique paper that summarizes detailed clinical and pathological findings of a large series of a rare disease. The clinical information presented in this paper is more plausible than previous views and is useful for accurate diagnosis and understanding of the disease.

## 1. Introduction

Castleman disease (CD) is a rare lymphoproliferative disease, consisting of several subtypes. Each subtype of CD has a different etiology, clinical manifestations, and histology. According to the number of enlarged lymph node regions, CD is divided into two major types: unicentric and multicentric. Unicentric CD (UCD) involves only one lymph node area and is often asymptomatic or mildly symptomatic, whereas multicentric CD (MCD) is associated with systemic symptoms and extensive lymph node involvement. UCD is further divided into two subtypes based on their histological characteristics: hyaline vascular (HV) type and plasma cell (PC) type. The HV type is characterized by increased lymphoid follicles with regressed germinal centers, hyalinized vessels, and hypervascularity within the interfollicular area. The PC type is characterized by hyperplastic germinal centers with sheet-like proliferation of plasma cells within the expanded interfollicular area. It has been reported that most cases of UCD are of the HV type (74.4–91.4%) [1,2,3,4].

CD was first reported in 1954 by Castleman et al. as a case of a 40-year-old man with a large mediastinal mass which turned out to be lymph node hyperplasia revealing follicles with hyalinized foci [5]. Subsequently, Castleman reported a series of 13 patients with enlarged mediastinal lymph nodes having histological features that were similar to those of currently defined HV-UCD [6]. HV-UCD is considered to be a histologically and clinically homogeneous entity, lacking systemic symptoms, and is usually curable with excision of local lesions. Keller and Castleman et al. reported that the most commonly affected sites in HV-UCD were the mediastinum and lung hilum (90.5%), neck (6.8%), retroperitoneum (1.4%), axilla (1.4%), and pelvis (1.4%) [1]. In the years when Castleman and Keller reported the disease, chest radiography was the main diagnostic modality, so the percentages of mediastinum and lung hilum lesions may have been higher than they actually were.

UCD can occur at any age and occurs equally in men and women, with a mean age at presentation of approximately 33.8–46.0 years [3,7,8,9,10]. In a systematic review of 235 cases of UCD it was found that the most common sites of involvement included the chest (28.9%), neck (23.4%), abdomen (20.9%), and retroperitoneum (16.6%) [8]. However, the included data in this review were from different periods and countries and may differ from the actual data. Another article, which was a case series, reported that in 43 patients with UCD (32 HV-CD and 11 PC-CD), the sites found to be commonly involved, in decreasing order of frequency, were the abdomen (39.5%), neck (23.3%), mediastinum (16.3%), and lung hilum (7.0%), with limited involvement of lymph nodes in the axilla and groin [4]. There are no studies to date that have exclusively reported data on HV-UCD. Thus, in the present study we have carried out a detailed clinical and pathological evaluation of 38 cases of HV-UCD and presented a comprehensive description of the obtained findings.

## 2. Materials and Methods

### 2.1. Patient Selection

The study protocol was approved by the Institutional Review Board of Okayama University, Okayama, Japan. We used opt-out to obtain consent for participation in this study. A total of 38 Japanese patients with HV-UCD were retrospectively identified and included in this study. All cases were retrieved from surgical pathology consultation files from the Department of Pathology of Okayama university between the years 2001–2020. In all cases, the lesions were localized in a single region. Immunostaining for CD20, CD3 and Ki-67 was performed in all these patients, and lymphoma was ruled out. Human herpes virus type 8 infectious status was not examined in this study population. None of the patients had autoimmune diseases or used corticosteroids or immunosuppressants. Other diseases with histopathological features overlapping with those of UCD were also excluded according to the guidelines of Frits et al. [11]. All the included patients had undergone surgery for total resection of the lesion.

### 2.2. Diagnosis of HV-UCD

The diagnosis of HV-UCD was made in postoperative surgical specimens and diagnosed by several pathologists at the department of pathology case review meeting. The pathological diagnosis of CD was established according to Keller’s criteria [1]. All cases satisfied the following histologic criteria: (1) proliferation of follicle-like structures, (2) regressed germinal center with radially penetrating capillaries, (3) extensive capillary proliferation with obliteration of lymphatic sinuses, and (4) perivascular hyalinization in both the germinal center and interfollicular area.

## 3. Results

### 3.1. Clinical Findings

The demographic and clinical characteristics of HV UCD patients are summarized in Table 1 and Table 2 provides detailed clinical information for all cases.

Of the 38 included patients, 20 were men and 18 were women with a mean age of 42.8 years (range, 12–76 years) at the time of diagnosis. Majority (24/38, 63.2%) of the patients were aged <50 years. Comorbidities included hyperlipidemia (3/38, 7.9%), asthma (2/38, 5.3%), and type 2 diabetes mellitus (1/38, 2.6%), as well as a history of malignant or infectious diseases that led to implementation of imaging studies for the diagnosis.

Patients who presented with subcutaneous masses or superficial lymph node swellings were aware of the mass or had localized pain, while most patients with lesions involving the deeper sites were asymptomatic. However, a few patients with retroperitoneal or pelvic masses complained of abdominal discomfort or back pain (2/38, 5.3%). Notably, in one case, the presence of paraneoplastic pemphigus led to the discovery of a lymph node lesion. The patient had oral erosions for two years prior to the diagnosis of HV-UCD. Subsequently, shortness of breath and coughing appeared. After a thorough examination, the patient was diagnosed with paraneoplastic pemphigus (PNP) and bronchiolitis obliterans (BO). Plasmapheresis and oral corticosteroids in a dose of 0.5 mg/kg were started along with resection of the lesion of HV-UCD, resulting in improvement of mucosal symptoms. The mucosal symptoms relapsed once during the tapering of steroids, but the PNP has been in remission since then with the maintenance dose of oral corticosteroids monotherapy (0.1 mg/kg). The BO has yet to go into remission but is well controlled with oral corticosteroids and has not had rapid exacerbations. The patient is still alive 20 years after the diagnosis of HV-UCD.

In 13 patients, lesions were incidentally found during diagnostic investigations for other comorbidities such as malignancy, gynecologic disease, or infection (13/38, 34.2%), whereas some cases were discovered as incidental findings during routine checkups (9/38, 23.7%).

The most common site of involvement was the abdominal cavity (13/38, 34.2%), with lesions of mostly mesenteric origin (7/13). This was followed by mediastinum (9/38, 23.7%), retroperitoneum (6/38, 15.8%), subcutaneous tissues of the back, buttocks, or extremities (4/38, 10.5%), axillary lymph nodes (2/38, 5.3%), cervical lymph nodes (2/38, 5.3%), hilar lymph nodes (1/28, 2.6%), and kidney (2.6%). The kidney mass was found within the parenchyma of the lower pole.

^18^F-fluorodeoxyglucose positron emission tomography-computed tomography (^18^F-FDG PET-CT) results could be obtained for the following seven patients: patient number 2, 7, 8, 25, 27, 28, 37. The mean maximum standardized uptake value (SUV-max) was 4.8 (range 2.5–9.1). In all these patients, the identical lesions that had ^18^F-FDG accumulation were surgically resected. There were no other sites showing ^18^F-FDG accumulation other than the resected lesions.

Preoperative differential diagnoses besides HV-UCD included gastrointestinal stromal tumor, solitary fibrous tumor, neurogenic tumor, lymphoma, metastatic carcinoma, and sarcoma in the abdominal cavity; paraganglioma, lymphoma, and sarcoma, in the retroperitoneum; thymoma, thymic carcinoma, neurogenic tumor, embryonal carcinoma, teratoma, and lymphoma, in the mediastinum.

The mean maximum diameter of the evaluated masses was 4.8 cm (range 1.7–11.0 cm). The mean diameters of the lesions in the abdominal cavity, retroperitoneum, and mediastinum were 4.1 cm (range 2.0–6.5), 5.8 cm (range 3.0–11.0), and 4.8 cm (range 1.7–9.0), respectively. All patients underwent total resection of the lesions, and none of them received chemotherapy or radiotherapy. Clinical follow-up was carried out in 17 cases, and no recurrences were reported. The mean follow-up period was 72.7 months. There were no abnormal hematological findings such as anemia, increased inflammatory markers, or hypergammaglobulinemia in any of the patients.

### 3.2. Histological Findings

**Figure 1:**
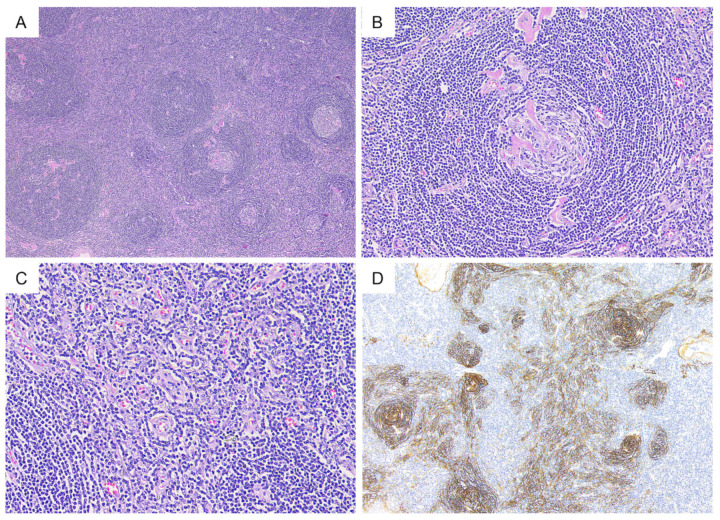
Representative histological findings in hyaline vascular type unicentric Castleman disease. (**A**) (H&E); hyperplasia of follicular structures. (**B**) (H&E); atrophic germinal center with penetrating hyalinized blood vessels and concentric mantle zones, which is a so-called “lollipop” appearance. (**C**) (H&E); extensive capillary proliferation in the interfollicular area was also observed. (**D**) (CD21); CD21 staining highlights prominent follicular dendritic cell meshwork.

**Figure 2:**
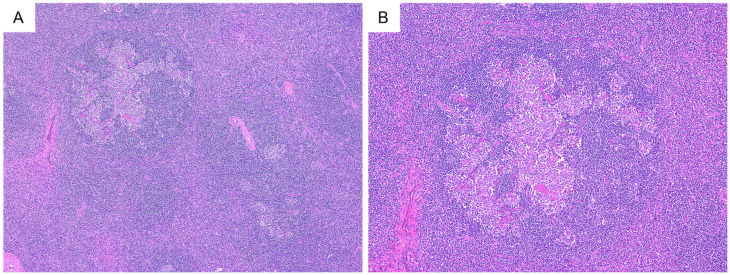
Progressive transformation of germinal centers-like structure. (**A**,**B**) (H&E); altered follicle structures with expanded mantle zones are shown.

Representative histological findings of the included cases are presented in Figure 1. HV-UCD is characterized by one or more atrophic germinal centers with small lymphocytes present concentrically around them. Radially penetrating capillaries or branched hyalinized vessels are often observed in atrophic germinal centers. In the interfollicular area, extensive capillary proliferation was observed. Plasmacytes and eosinophils were found sporadically, and no aggregation or sheet-like proliferation of plasma cells was observed. Altered follicle structure with expanded mantle zone (progressive transformation of germinal center-like pattern) was observed in four cases (Figure 2).

**Figure 3:**
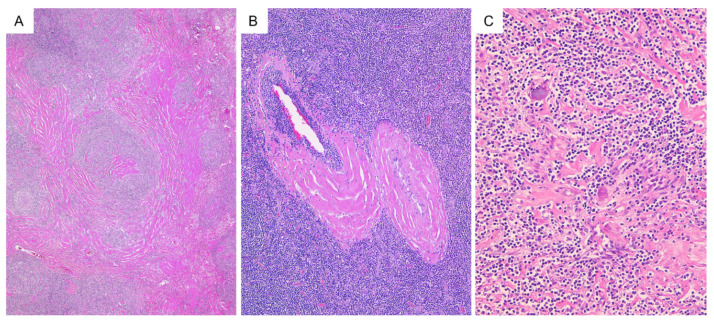
Hyalinization and calcification in hyaline vascular type unicentric Castleman disease. (**A**) (H&E); broad hyalinized collagen fibers spreading throughout the lesion. (**B**) (H&E); thick hyalinized collagen fibers surrounding large blood vessels. (**C**) (H&E); calcium deposition in hyalinized area.

The degree of hyalinization was variable. Thick hyalinized collagen fibers were seen in the interfollicular area surrounding large blood vessels while broad hyalinized collagen fibers spreading throughout the lesion were also often observed (31/38, 81.6% and 15/38, 39.5%, respectively) (Figure 3). This broad hyalinized area was associated with calcification in nine patients (23.7%).

**Figure 4:**
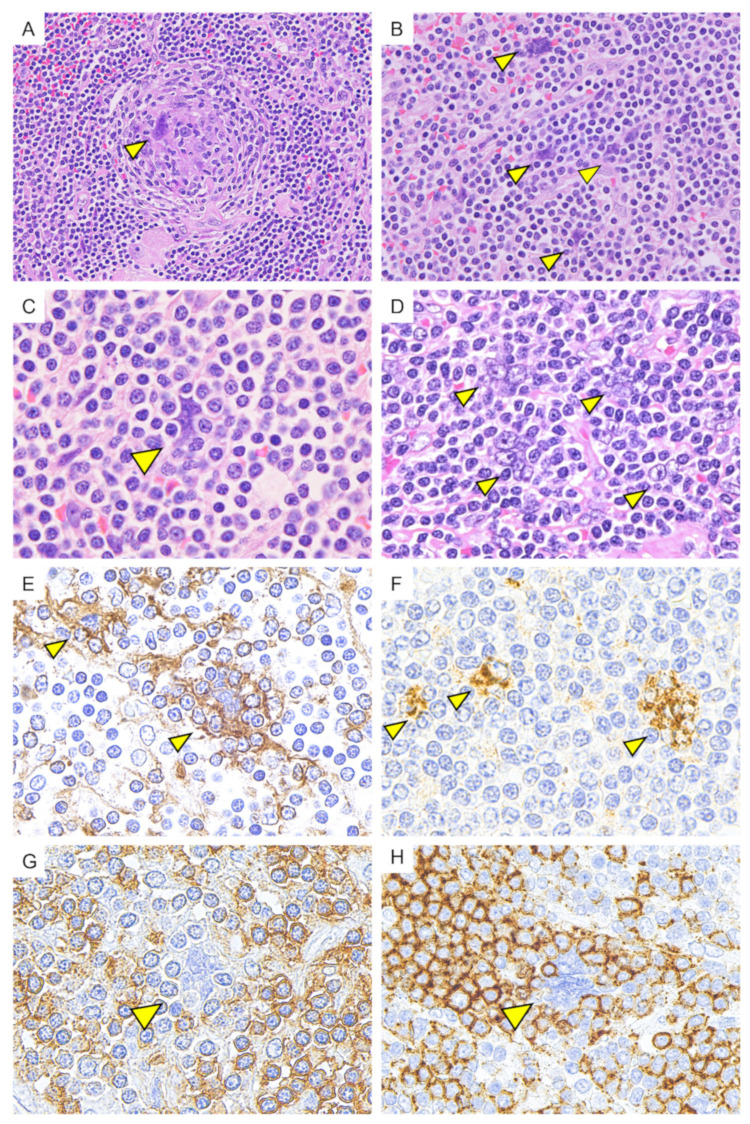
Multinucleated giant cells resembling Warthin–Finkeldey cell (indicated by an arrowhead). (**A**,**B**) (H&E); Multinucleated giant cells resembling Warthin–Finkeldey cell were observed in germinal center area and interfollicular area. (**C**,**D**) (H&E); the morphology of multinucleated giant cells showed some variation, but the nuclei with a single distinct nucleolus were arranged in a grape-like cluster. (**E**) (CD21); the cytoplasm of multinucleated giant cells was positive for CD21. The cytoplasmic border was indistinct with lymphocytes entrapment. (**F**) (CXCL13); CXCL13 was positive in the cytoplasm of multinucleated giant cells. (**G**) (CD43) and (**H**) (CD20); small lymphocytes entrapped in the cytoplasm of multinucleated giant cells are negative for CD43 and positive for CD20.

In the follicular and interfollicular area, multinucleated giant cells resembling Warthin–Finkeldey cell were observed in occasional cases (9/38, 23.7%) (Figure 4). These multinucleated giant cells were observed in the germinal center and interfollicular area. These multinucleated cells were positive for CD21 and CXCL13 in the cytoplasm, and negative for CD23, CD20, CD3, CD43, CD30, and CD68. In CD21 staining, the cytoplasmic border was indistinct, and the cells appeared to trap the surrounding small lymphocytes. These small lymphocytes surrounding the multinucleated cell were negative for CD43 and positive for CD20, suggesting B cells.

## 4. Discussion

In this study, we present detailed clinical data and pathological findings of 38 histologically well-defined HV-UCD cases. This is a unique study considering the fact that exclusive reports on HV-UCD are scarce in the existing literature and as such detailed evaluation of multiple cases of HV-UCD will add immensely to the current knowledge of this entity.

### 4.1. Clinical Characteristics

Data on age, sex, and symptoms were similar to those in the previous reports [3,7,8,9,10]. As discussed earlier, HV-UCD has been thought to occur predominantly in the mediastinal region, but our study revealed that HV-UCD most common involvement site was in the abdominal cavity. Given the advances in imaging modalities [12], the results of the present study may be closer to the actual lesion distribution of HV-UCD.

While malignant tumors (rectal cancer and uterine cancer), benign tumors (uterine leiomyoma, ovarian benign serous tumor, and mature teratoma of the mediastinum), and inflammatory diseases (ascending colon diverticulitis and ischemic enteritis) were present in nine patients, the relationship between these diseases and HV-UCD has been unclear. In severe infections and autoimmune diseases, the adjacent lymph nodes may show reactions such as follicular hyperplasia, hypervascularization, and plasma cell proliferation in the enlarged interfollicular area. However, the histological features of HV-UCD, such as hyalinization of the blood vessels and atrophy of the germinal center, are uncommon in these diseases, so histological differentiation is not considered to be a problem. The patients included in this study did not have any underlying disease that could exhibit histology similar to HV-UCD.

One of our patients had PNP and BO in association with CD. A previous report has shown that CD is one of the most common causes of tumor-associated pemphigus, along with non-Hodgkin’s lymphoma and other hematologic tumors [13,14]. BO is a serious manifestation of PNP and is one of the leading causes of mortality. Although the frequency varies among reports, it has been reported that about 30% of PNP is associated with BO [15,16], with a higher frequency in children and adolescents with CD. In one report, the frequency of bronchiolitis obliterans was 93% in a series of 28 patients with PNP, which included 15 children and adolescents with CD [17]. Since autoantibodies produced by tumor cells are responsible for the pathogenesis of mucocutaneous lesions, removal of solid tumors and remission of hematologic tumors by chemotherapy are critical for improvement of PNP [18]. Complete resection of localized Castleman’s disease may result in significant improvement or complete remission of the condition [19]. However, it takes several years after surgery to achieve remission, and during that time, serious complications such as respiratory failure can occur.

In this study, four cases of subcutaneous masses, which are considered rare, were also included [20]. One of this was a mass arising in the kidney, which is an extremely rare location for HV-UCD lesions [21]. Other unusual sites such as the orbits, nasopharynx, lungs, and spleen have also been reported in literature [22,23,24,25]. Thus, it is important to keep in mind that these unusual sites might also be involved, while making a diagnosis of HV-UCD.

There have been several reports of ^18^F-FDG avid UCD lesions [3,4,26,27,28,29]. In these reports, FDG uptake was moderately increased in UCD, and SUVs were generally less than those observed in lymphoma. Our results were also consistent with this.

### 4.2. Histological Characteristics

HV-UCD is preferably diagnosed by excisional biopsy of the enlarged lymph nodes. Although there are several diseases that show CD-like histopathological features such as unusual morphological variants of follicular lymphoma, angioimmunoblastic T-cell lymphoma, these usually present with generalized lymphadenopathy and are rarely confused with UCD [11].

The finding of altered follicular structure with expanded mantle zone, as shown in Figure 2, may also be seen as a progressive transformation of germinal centers. In the present study, calcification was found in 23.7% of lesions. Various patterns of calcification have been reported within the lesion in past reports [3,30,31,32,33]. Calcification and extensive hyalinization may indicate that the lesion is indolent.

In this study, multinucleated giant cells resembling Warthin–Finkeldey cells were observed in the germinal center or interfollicular area in nine cases. Although Warthin–Finkeldey cells are commonly seen in Kimura disease, a rare chronic inflammatory disease with unclear etiology, these are not unique to Kimura disease and are also observed in measles and a variety of reactive or neoplastic lymphoid conditions. In the present study, these multinucleated giant cells were positive for the follicular dendritic cell (FDC) markers such as CD21 and CXCL13, and were considered to be identical to those referred to as dysplastic FDCs in previous reports [34,35,36,37,38]. It has been reported that FDC sarcoma may occur in lymph nodes in the same region subsequently to UCD [37,38], suggesting that dysplastic FDCs may be a primary driver of UCD pathogenesis.

### 4.3. Future Prospects

The etiology of HV-UCD has been unclear since the disease was first reported over 60 years ago. Although the lineage of cells responsible for the pathogenesis of UCD has not been identified, several studies have hypothesized that a neoplastic stromal cell population may be the precursor of UCD [39,40,41,42,43,44]. Recently, Li et al. found recurrent PDGFRB N666S mutations in UCD cases, and they also investigated the cell of origin using BaseScope, a mutation-specific RNA in situ hybridization assay. Their results suggested that the PDGFRB N666S mutations were confined to non-hematopoietic stromal cells in UCD, and it is likely that they play an important role in UCD pathogenesis [45].

With this latest body of evidence, we have a better understanding of the pathogenesis of HV-UCD. However, owing to the rarity of the disease, it is necessary to accumulate further evidence with a focus on genetic analysis in the future.

## 5. Conclusions

In conclusion, our study provides up-to-date clinical and pathological findings of patients with HV-UCD. The detailed clinical information presented in this paper is more plausible than previously thought and is useful for accurate diagnosis and understanding of the disease. Comprehensive genetic analysis of histologically well-defined HV-UCD patients is required as a next step in this area of study to clarify the pathogenesis of the disease.

## Figures and Tables

**Table 1:** Demographic and Clinical Features of Patients with HV-type UCD.

	n = 38
Age (year)	
mean ± SD (range)	42.8 ± 17.8 (12–76)
<50, n (%)	24 (63.2)
≥50, n (%)	14 (36.8)
Sex (M:F)	20:18
Symptom, n (%)	
None	27 (71.1)
Local pain	2 (5.3)
Awareness of the mass	7 (18.4)
Abdominal discomfort	1 (2.6)
Paraneoplastic pemphigus	1 (2.6)
Site, n (%)	
Abdominal cavity	13 (34.2)
Mesentery	7 (18.4)
Omentum	2 (5.3)
Lesser omentum	1 (2.6)
Abdominal wall	1 (2.6)
Pelvis	2 (5.3)
Mediastinum	9 (23.7)
Posterior	4 (10.5)
Anterior	2 (5.3)
Superior	2 (5.3)
Middle	1 (2.6)
Retroperitoneum	6 (15.8)
Lymph node	5 (13.2)
Axillary Lymph node	2 (5.3)
Cervical Lymph node	2 (5.3)
Hilar Lymph node	1 (2.6)
Soft tissue	4 (10.5)
Kidney	1 (2.6)
Tumor diameter (cm) mean ± SD (range)	
Total	4.8 ± 2.2 (1.7–11.0)
Abdominal	4.1 ± 1.4 (2.0–6.5)
Retroperitoneum	5.8 ± 3.0 (3.0–11.0)
Mediastinum	4.8 ± 2.3 (1.7–9.0)
Soft tissue	4.3 ± 1.2 (3.0–5.0)
Laboratory findings (mean ± SD)	
WBC (/µL) (n = 22)	5467 ± 1663
Hb (g/dL) male (n = 13)	15.0 ± 1.0
Hb (g/dL) female (n = 9)	13.4 ± 1.4
Plt (×10^4^/µL) (n = 22)	26.8 ± 7.1
TP(g/dL) (n = 21)	7.3 ± 0.4
Alb (g/dL) (n = 21)	4.4 ± 0.5
CRP (mg/dL) (n = 20)	0.2 ± 0.2
LDH (U/L) (n = 22)	184 ± 53.5

Abbreviations: SD; standard deviation, WBC; white blood cell, Hb: hemoglobin, Plt; platelet, TP; Total protein, Alb; albumin, CRP; C-reactive protein, LDH; Lactate Dehydrogenase. Normal ranges: WBC 3900–9800/µL, Hb 13.4–17.6 g/dL (male) 11.3–15.2 g/dL (female), Plt 12.7–35.6 × 104/µL, TP 6.7-8.3 g/dL, Alb 3.7–5.2 g/dL, CRP 0.00–0.30 mg/dl, LDH 119-229 U/L.

**Table 2:** Detailed of clinical findings.

Case	Age	Sex	PMH and Comorbidities	Site	Symptoms	Modality	Tumor Diameter (cm)	Preoperative Diagnosis
1	29	M	None	Retroperitoneum	None	Lumbar X-ray for lumbar sprain	3.6	―
2	53	M	Colonic diverticulitis	Abdominal mass (mesoduodenum)	None	Abdominal US for diverticulitis	3.0	―
3	61	F	Hypothyroidism, Hyperlipidemia	Retroperitoneum	None	Abdominal CT for hematuria	4.1	UCD, paraganglioma, Liposarcoma, Leiomyosarcoma
4	47	M	Ischemic enteritis	Abdominal mass (mesoduodenum)	None	Abdominal CT for ischemic enteritis	2.0	GIST, Leiomyoma, Neurogenic tumor, SFT
5	38	F	None	Mediastinum (middle)	None	CT for high serum SCC antigen	6.0	Teratoma, Thymoma
6	39	M	None	Abdominal mass (pelvis)	Back pain	CT for back pain	5.0	―
7	36	M	Hyperlipidemia, Chronic gastritis, Asthma	Abdominal mass (mesojejunum)	None	Abdominal US for mild liver dysfunction	6.5	GIST, Neurogenic tumor, UCD
8	28	M	Type 2 DM, Asthma	Mediastinum (anterior)	None	Screening chest X-ray	9.0	Thymoma, Thymic carcinoma, Embryonal tumor
9	16	M	None	Mediastinum (posterior)	None	School screening chest X-ray	5.0	―
10	58	M	Rectal cancer (post-therapeutic state)	Abdominal mass (transverse mesocolon)	None	Follow-up CT for rectal cancer	2.5	Cancer dissemination
11	74	M	Post cholecystectomy	Mediastinum (anterior)	None	CT for transient loss of consciousness	2.6	Thymoma
12	15	M	None	Mediastinum (superior)	None	School screening chest X-ray	5.0	UCD, neurogenic tumor
13	12	F	None	Soft tissue of buttocks (subcutaneous)	Subcutaneous mass, Local pain	MRI for subcutaneous mass	5.0	Chronic expanding hematoma
14	39	F	None	Abdominal mass (omentum)	None	Abdominal US for regular checkup	5.0	UCD
15	50	F	Post splenectomy and cholecystectomy	Retroperitoneum	Abdominal discomfort for the past two years	Abdominal CT for abdominal discomfort	7.6	Lymphoma, Sarcoma, UCD
16	28	F	Mediastinal teratoma (post-operative state)	Retroperitoneum	None	Abdominal US for transient fever	11.0	―
17	76	F	None	Retroperitoneum	None	Abdominal US for regular checkup	3.0	UCD, Paraganglioma
18	23	F	Amenorrhea (under hormonal therapy)	Abdominal (sigmoid mesocolon)	None	Abdominal US for amenorrhea	5.0	Lymphoma, Sarcoma
19	31	M	None	Axillary LN	LN swelling for the past 10 years	CT for thorough medical checkup	3.6	Reactive lymphadenopathy
20	45	F	Uterine leiomyoma, Ovarian benign serous tumor	Abdominal mass (mesoileum)	None	Abdominal CT for ovarian lesion	4.5	GIST
21	63	F	Endometrial carcinoma (post-operative state)	Mediastinum (posterior)	None	Follow-up CT for endometrial carcinoma	1.7	UCD
22	47	M	None	Mediastinum (posterior)	None	CT for thorough medical checkup	6.5	UCD, neurogenic tumor
23	52	M	None	Axillary LN	LN swelling for the past three years	CT for LN swelling	7.7	Lymphoma
24	69	F	None	Kidney (inferior pole of right kidney)	None	CT for thorough medical checkup	2.0	RCC, Leiomyoma, Angiomyolipoma
25	75	F	Hyperlipidemia, Arteriosclerosis	Abdominal mass (abdominal wall)	None	Screening CT for coronary arteriosclerosis	2.3	UCD, NET, GIST, SFT
26	46	M	None	Soft tissue of back (subcutaneous)	Subcutaneous mass for the past 20 years	CT and MRI for subcutaneous mass	4,0	Sarcoma
27	20	F	None	Mediastinum (superior)	None	Screening chest X-ray	4.8	UCD, Lymphoma, Neurogenic tumor
28	48	F	Uterine leiomyoma	Abdominal mass (sigmoid mesocolon)	None	Abdominal CT for genital bleeding	3.4	UCD
29	53	M	None	Abdominal (omentum)	None	CT for thorough medical checkup	5.8	―
30	20	M	None	Retroperitoneum	PNP, BO	Screening CT	5.6	UCD
31	38	F	Uterine leiomyoma, Para fallopian tube cyst	Abdominal mass (pelvis)	None	Abdominal CT for genital bleeding	4.0	―
32	38	M	None	Abdominal mass (lesser omentum)	None	Abdominal US for mild liver dysfunction	4.0	―
33	53	F	None	Cervical LN	LN swelling for the past 10 years	CT for LN swelling	6.3	Reactive lymphadenopathy, Lymphoma
34	14	M	None	Soft tissue of left upper arm (subcutaneous)	Subcutaneous mass	MRI for subcutaneous mass	3.0	Soft tissue tumor
35	52	F	None	Soft tissue of right upper arm (subcutaneous)	Subcutaneous mass	MRI for subcutaneous mass	5.0	―
36	47	M	None	Mediastinum (posterior)	None	CT for thorough medical checkup	2.6	GIST, Neurogenic tumor
37	28	M	None	Hilum LN	None	Screening chest X-ray	3.6	UCD, SFT, Carcinoid tumor
38	65	F	None	Cervical LN	None	CT for thorough medical checkup	1.6	IgG4-related disease, Lymphoma

Abbreviations: PMH; past medical history, DM; diabetes mellitus, LN; lymph node, SCC; squamous cell carcinoma, US; ultrasonography, CT; computed tomography, MRI; magnetic resonance imaging, UCD; unicentric Castleman disease, GIST; gastrointestinal tumor, SFT; solitary fibrous tumor, RCC; renal cell carcinoma, NET; neuroendocrine tumor, PNP; paraneoplastic pemphigus, BO; bronchiolitis obliterans.

## Data Availability

Not applicable.
